# Polyphyllin I suppressed the apoptosis of intervertebral disc nucleus pulposus cells induced by IL-1β by miR-503-5p/Bcl-2 axis

**DOI:** 10.1186/s13018-023-03947-7

**Published:** 2023-06-28

**Authors:** Lei Yuan, Hui Miao, Heng Ding, Fan Zhang, Zhen-kai Lou, Xing-Guo Li

**Affiliations:** 1 Department of Orthopedics, The First Affiliated Hospital of Kunming Medical University, No. 295, Xichang Road, Wuhua District, Kunming City, 650031 Yunnan Province China; 2 Rehabilitation Department, Yantai Yuhuangding Hospital, Yantai, 264001 Shandong China

**Keywords:** Polyphyllin I, IL-1β, NPCs apoptosis, miRNA-503-5p, Bcl-2

## Abstract

**Background:**

There are no studies that have shown the role and underlying mechanism of Polyphyllin I (PPI)-mediated anti-apoptosis activity in nucleus pulposus cells (NPCs). The research aimed to evaluate the effects of PPI in interleukin (IL)-1β-induced NPCs apoptosis in vitro.

**Methods:**

Cell Counting Kit-8 (CCK-8) assay was used to detect cell viability, and cell apoptosis was evaluated by double-stained flow cytometry (FITC Annexin V/PI). The expression of miR-503-5p was quantified by real-time quantitative PCR (qRT-PCR), and the expression of Bcl-2, Bax, and cleaved caspase-3 was quantified by Western blot. Dual-luciferase reporter gene assay was used to detect the targeting relationship between miR-503-5p and Bcl-2.

**Results:**

PPI at 40 μg·mL^−1^ markedly promoted the viability of NPCs (*P* < 0.01). Also, PPI inhibited apoptosis and reduction in proliferative activity induced by IL-1β in the NPCs (*P* < 0.001, 0.01). PPI treatment significantly inhibited the expression of apoptosis-related protein Bax, cleaved caspase-3 (*P* < 0.05, 0.01), and enhanced the level of anti-apoptotic protein Bcl-2 (*P* < 0.01). The proliferative activity of NPCs was significantly decreased and the apoptosis rate of NPCs was increased under IL-1β treatment (*P* < 0.01, 0.001). Moreover, miR-503-5p was highly expressed in IL-1β-induced NPCs (*P* < 0.001). Furthermore, the effect of PPI on NPCs viability and apoptosis in IL-1β treatment was dramatically reversed by the overexpression of miR-503-5p (*P* < 0.01, 0.01). The targeted binding of miR-503-5p to the 3'UTR of Bcl-2 mRNA was confirmed by dual-luciferase reporter gene assays (*P* < 0.05). In further experiments, compared with miR-503-5p mimics, the effects of PPI on IL-1β-induced NPCs viability and apoptosis were greatly reversed by the co-overexpression of miR-503-5p and Bcl-2 (*P* < 0.05, 0.05).

**Conclusion:**

PPI suppressed the apoptosis of intervertebral disk (IVD) NPCs induced by IL-1β via miR-503-5p/Bcl-2 molecular axis.

## Background

Intervertebral disk degeneration (IVDD) is one of the major causes of low back pain, seriously endangering public health and bringing a huge economic burden around the world [1]. However, the molecular mechanism of IVDD has not been clearly elucidated. Multiple in vivo and in vitro factors may cause IVDD, such as genetic factors, intervertebral disk dystrophy, immunological factors, matrix metalloproteinases, inflammatory mediators, extracellular matrix (ECM) factors, apoptosis, and mechanical overload [2]. In disk degeneration, there is a decrease in cell numbers and altered cell function in the nucleus pulposus (NP), resulting in an imbalance between matrix synthesis and degradation [3, 4]. More evidence showed that IVDD occurrence has been generally considered to be associated with excessive apoptosis of NPCs [5]. Thus, inhibiting NPCs apoptosis may slow down the IVDD progression, which is of great significance to improve the quality of life of IVDD patients.

Chinese herbal medicine has a history of thousands of years in the treatment of multiple diseases. PPI, also called Chong Lou saponin I, a steroidal saponin, is a bioactive substance separated from the rhizome of Paris polyphyllin. Previous reports revealed that PPI possessed an anti-cancer effect in a variety of cancers by inhibiting the proliferation and growth of tumor cells [6–10]. For example, Long et al. reported that PPI promoted melanoma cells’ autophagy and apoptosis via PI3K/Akt/mTOR signaling pathway [11]. Tian et al. showed that PPI induces apoptosis and autophagy via modulating JNK and mTOR pathways in human acute myeloid leukemia cells [12]. Therefore, PPI is used as a possible anti-cancer drug candidate because of its pro-apoptotic role. However, Huang et al. found that PPI could attenuate apoptosis after ischemia/reperfusion injury in rats [13]. Moreover, Yang et al. reported that PPI eased apoptosis in myocardial cells and coronary artery disease (CAD) rat model [14]. These results indicate that PPI may play different roles under different pathological conditions. In fact, the beneficial effect of PPI on IVDD may be related to the significant inhibition of the secretion of inflammatory cytokines and the translocation of p65 [15, 16]. Relevant literature has confirmed that PPI has an anti-apoptotic effects to myocardial cells. However, its protective effects on NPCs and its mechanism of action are still unclear. In the current research, we sought to explore the underlying regulated mechanism of PPI on apoptosis of NPCs in vitro. The beneficial influences of PPI on NPCs were studied. The research supplemented the role of PPI in IVDD, providing a reference for further exploring the pathogenesis and treatment strategies of IVDD.

## Methods

### Source of tissue samples

The experimental protocol in this study was approved by the Ethics Committee of Kunming Medical University. All participants and their guardians were well-informed and signed the informed consent form prior to the study. From May 2021 to August 2021, the normal NP tissues were obtained from ten patients (mean age 25 ± 3 years) with idiopathic scoliosis classified as Pfirrmann grade I or II according to magnetic resonance imaging (MRI). All patients had no history of tumor, tuberculosis, diabetes, chronic infection, and autoimmune diseases (Table 1).

**Table 1:** Demographic details of the samples considered for this study

Group	Age, years	Sex	Disk level	Pfirrmann grade
ND	25	Male	L4-L5	2
ND	24	Female	L4-L5	1
ND	31	Male	L4-L5	1
ND	26	Male	L4-L5	2
SD	29	Female	L2-L3	2
SD	24	Female	L3-L4	2
SD	23	Female	T12-L1	2
SD	21	Female	T12-L1	2
SD	23	Male	L3-L4	2
SD	24	Female	L1-L2	2

*ND* normal disk; *SD* scoliotic disk

### Cell culture, grouping and transfection

The tissue material was quickly separated under sterile conditions. Then, the annulus fibrosus and excess parts were removed, and nucleus pulposus tissues were retained. After rinsing with D-Hank's solution, the NP tissues were cut into cubes measuring approximately 1mm^3^. Then, the NP tissues were digested in 0.25% trypsin for 15 min and 0.025% type II collagenase for 8 h at 37 °C. Washed the cells three times with D-Hank’s solution, centrifuged them (1000 rbm, 5 min), and discarded the supernatant. The cell pellet was resuspended in Dulbecco's modified Eagle's medium/F12 (DMEM/F12) (GIBCO, 12,400–024) supplemented with 10% fetal bovine serum (FBS) (GIBCO, 10,099–141), 1% glutamine (GIBCO 1894153), 100 U/mL of penicillin and 100 μg/mL of streptomycin (GIBCO) at 37 °C and 5% CO_2_. Medium was changed three times a week. Third-generation NPCs were used for in vitro experiments.

NPCs were divided into the negative control (NC) group, PPI concentration gradient group, IL-1β group, IL-1β + PPI group (40 μg/mL), IL-1β + PPI + miR-503-5p overexpression group, and IL-1β + PPI + miR-503-5p overexpression + Bcl-2 overexpression group. The treatment concentration of IL-1β was 10 ng/mL.

Transfection experiments were performed using Lipofectamine 2000 according to the manufacturer's instructions (Invitrogen). The cells were treated according to the experimental grouping described above. The overexpression and knockdown experiments of miR-503-5p were that miR-503-5p mimic and inhibitor were transfected into NPCs, respectively. Moreover, miRNA scramble was transfected as a negative control. Bcl-2 overexpression treatment transfected the pcDNA3.1 recombinant vector that overexpressed Bcl-2, and then set up a control empty plasmid group. Three replication wells were set up for each transfection experiment. After 48 h, qRT-PCR was used to detect the transfection efficiency of each group.

### CCK-8 assay

NPCs were digested with 0.25% pancreatic enzyme and inoculated into 96-well plates with 5000 cells per well and three wells per group. The cells were screened by CCK-8 kit (Beyotime, Shanghai, China) after stable adherence. Subsequently, the blank group (containing culture medium and CCK-8 solution), the control group (containing untreated cells, culture medium and CCK-8 solution), and the experimental subgroup (containing cells, culture medium and CCK-8 solution and PPI) were set up. Then, the experimental group was divided into seven subgroups with the concentration of 5, 10, 20, 40, 60, 80, and 100 μg·mL^−1^ of PPI. Then, 10 μL of CCK-8 reagent was added into each well, and the OD value was measured 2 h later at wavelength of 450 nm with a microplate reader after changing the medium. The survival rate (%) = (absorbance value of experimental group − absorbance value of blank control group)/(absorbance value of control group − absorbance value of blank control group) × 100%, and then calculated results. Subsequent experiments were conducted with concentrations of 40 μg·mL^−1^ of PPI and cell viability was detected with CCK-8 assay.

### Flow cytometry analysis

Cells were washed with phosphate-buffered saline (PBS), followed by detachment with trypsinization and centrifugation to collect cells. 300 μL binding buffer was used to resuspend cells and added 10 μL annexin V-FITC solution gently mix well. The cells were incubated in dark at room temperature for 15 min, then 5 μL PI dye and 200 μL of the binding solution were added. Cell apoptosis was detected by flow cytometry (BD, Franklin lakes, New Jersey, USA).

### Quantitative real-time PCR

Total RNA in NP tissues or cells were extracted using TRIZOL agent (Lifetech 15,596,026), quantified for RNA concentration using an ultraviolet spectrophotometer, and reversely transcribed into cDNA using PrimeScript RT kit. The appropriate amount of cDNA was used as the template for PCR primers, which was designed using the software Primer 5.0 (Table 2). The amplification program was as follows: initial denaturation at 95 °C for 10 min, followed by 50 cycles of denaturation at 95 °C for 15 s, annealing at 60 °C for 35 s and extension at 72 °C for 10 s. miR-503-5p used U6 as an internal reference, and mRNA-Bcl-2 used GADPH as an internal reference. The relative miRNA expression was calculated using the 2^−ΔΔCT^ methods.

**Table 2:** Primer sequence

Gene	Primers (5′–3′)
BCL2	F: 5′-ACGCCTACTACCTTCAAGC-3′
	R: 5′-CCACTTGGAACATACCGAT-3′
miR-503	F: 5′-GTG-CAGGGTCCGAGGT-3′
	R: 5′-GCTAGCAGC-GGGAACAG-3′
GAPDH	F: 5′-AAAGGGTCATCATCTCTG-3′
	R: 5′-GCTGTTGTCATACTTCTC-3′
U6	F: 5′-CTCGCTTCG- GCAGCACA -3′
	R: 5′-AACGCTTCACGA -ATTTGCGT-3′

### Western blot analysis

Total proteins were extracted from cells a using RIPA buffer (Beyotime, Shanghai, China). Then, protein samples (30 μg per lane) were separated on 10% SDS-PAGE gels (Bio-Rad, USA) and transferred to a PVDF membrane (Millipore, USA). Sealed with PBS buffer containing 5% skim milk powder for 2 h, and added anti-Bcl-2 (1: 1000, Beyotime, Shanghai, China), anti-Bax (1∶2000, Beyotime, Shanghai, China), anti-cleaved caspase3 (1∶2000, Beyotime, Shanghai, China), anti-GAPDH (1∶2000, Beyotime, Shanghai, China) overnight at 4 °C, the PBST buffer was rinsed 3 times, 5 min each time. Next, Bcl-2, Bax, cleaved caspase3 and GAPDH secondary antibodies (sheep versus rabbit, Beyotime, Shanghai, China) were added, and incubated at 37 °C for 4 h. PBST buffer was rinsed for 3 times, and the gray value of target protein was analyzed by Image J.

### Dual-luciferase reporter gene assay

ENCORI database (http://starbase.sysu.edu.cn/index.php) was used to predict the target site of Bcl-2 3′UTR to bind to miR-503-5p. The sequences were cloned into a pmirGLO reporter vector, located downstream of the luciferase gene, to generate the recombinant vectors pmirGLO-Bcl-2-wt and pmirGLO-Bcl-2-mut. Besides, pmirGLO-Bcl-2-WT/Mut reporter vectors were severally co-transfected with miR-503-5p mimics or NC mimics into NPCs. After 48 h of transfection, the cells were collected and lysed, centrifuged for 3–5 min, and 5 µL supernatant was taken. The luciferase activities were determined with a dual-luciferase assay reporter system (Promega, Madison, WI, USA), according to the manufacturer’s instructions. Renilla luciferase fluorescence value was used as an internal reference.

### Statistical methods

All samples were in triplicate, and the experiment was repeated 3 times. Data were expressed in terms of mean and standard deviation. SPSS 20.0 software (IBM, USA) was used for statistical analysis, where one-way ANOVA was used for test of difference between multiple groups, and T test was used for test of difference between two groups. GraphPad Prism 9 (GraphPad, USA) software was used to draw relevant pictures of experimental data. A statistical *P* value < 0.05 was considered statistically significant.

## Results

### PPI treatment significantly attenuated IL-1β-induced apoptosis of NPCs

**Fig. 1:**
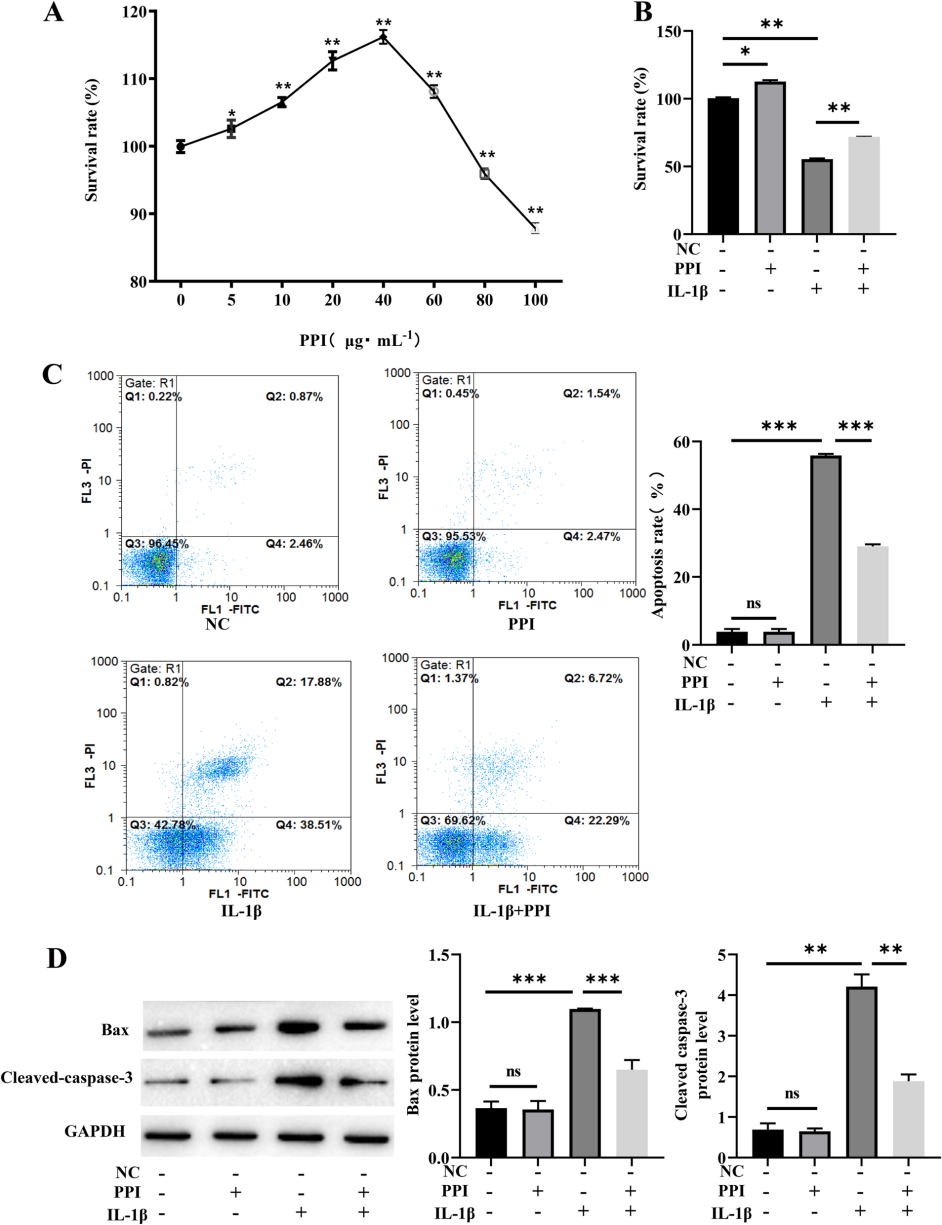
NPCs apoptosis induced by IL-1β was significantly inhibited by PPI. **A** Cells were cultured for 24 h with PPI at different concentrations, and cell survival rate was detected by the CCK-8 assay. **B** CCK-8 assay was used to detect the effects of PPI on the survival rate of NPCs. **C** Flow cytometry was used to detect the effects of PPI on the apoptosis induced by IL-1β of NPCs. **D** Western blot was used to detect the expression of Bax and cleaved caspases-3 (n = 1–2 IVDD patients/group with 3–4 samples/patient). Data are shown as means plus or minus a standard deviation. ns: not significant, *P < 0.05, **P < 0.01, and ***P < 0.001

The protection of PPI on NPCs was determined by CCK-8 assay. NPCs were treated with PPI (Fig. 1A) of indicated doses (5, 10, 20, 40, 60, 80, 100 μg/mL) for 24 h, and then CCK-8 assay was applied to determine cell growth. The results manifested that PPI treatment could promote NPCs proliferation in a dose-dependent manner. At the concentration higher than 40 μg/mL, the survival rate of NPCs was significantly reduced. So, the dose of 40 μg/mL PPI was selected as the effective and safe concentration for our experiments. As shown in Fig. 1B, IL-1β-induced cytotoxicity in NPCs and IL-1β (10 ng/mL) applied for 24 h significantly decreased cell viability (*P* < 0.05). Surprisingly, PPI markedly improved cell viability in IL-1β-stimulated NPCs. In addition, flow cytometric Annexin V/PI was used to assess the prevention of PPI on IL-1β-induced apoptosis. The results of flow cytometric analysis showed that apoptosis of NPCs significantly increased after IL-1β treatment. However, PPI treatment inhibited IL-1β-induced apoptosis of NPCs (Fig. 1C). Furthermore, the expressions of apoptosis-associated proteins were measured to understand the molecule mechanisms. As we expected, the levels of Bax and cleaved caspases-3 were significantly elevated in comparison with the NC group (*P* < 0.05). However, PPI treatment alleviated the levels of Bax and cleaved caspases-3 (Fig. 1D, *P* < 0.01, *P* < 0.001). All these findings indicated that PPI prevented IL-1β-induced NPCs apoptosis.

### PPI inhibited IL-1β-induced apoptosis via downregulation of miR-503-5p expression

**Fig. 2:**
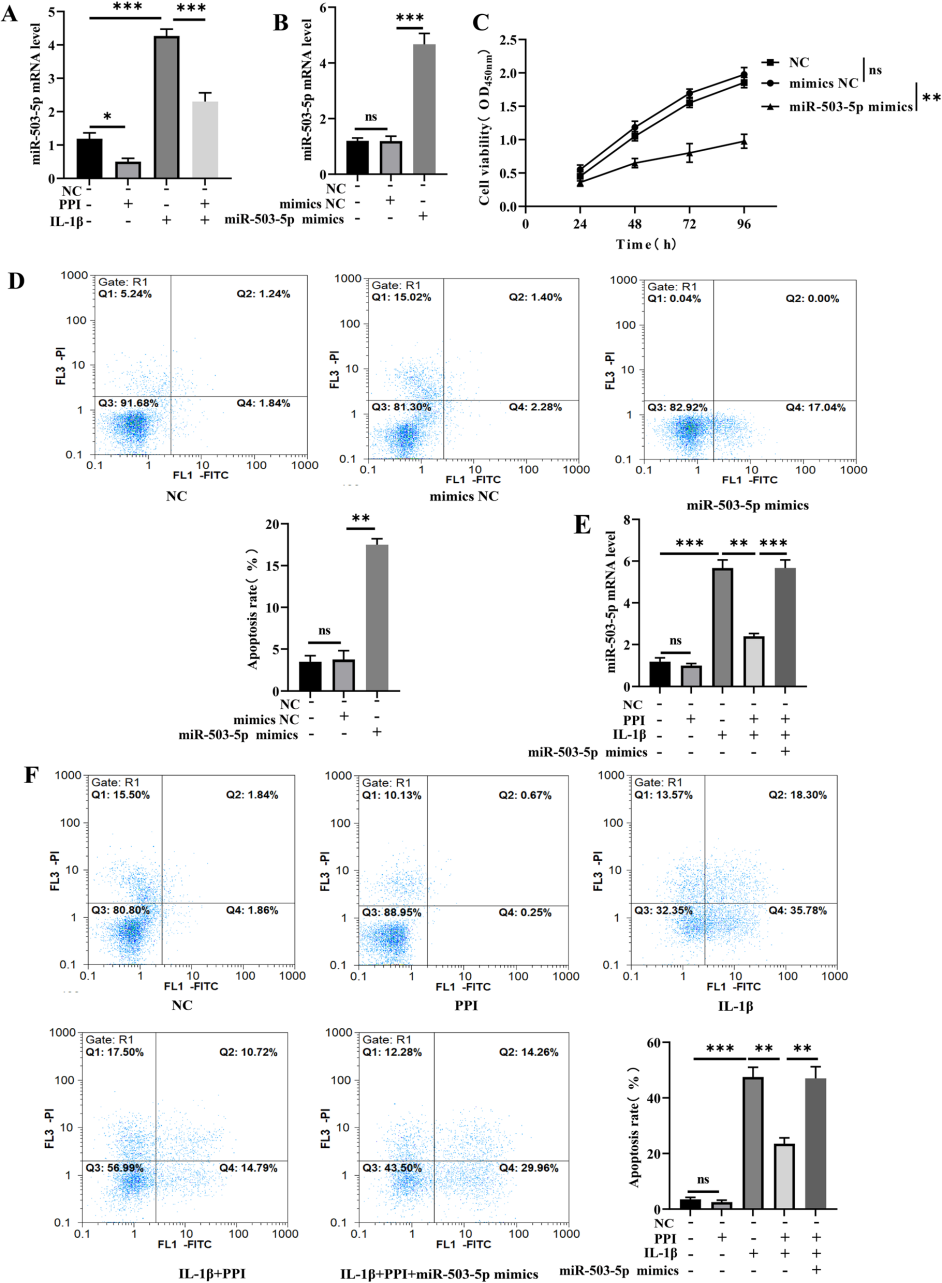
PPI dramatically inhibited IL-1β-induced NPCs apoptosis via downregulation of the expression of miR-503-5p. **A** qRT-PCR was applied for miR-503-5p quantification in the control, PPI, IL-1β, and IL-1β + PPI group. **B** qRT-PCR of miR-503-5p in NPCs with the miR-503-5p expression vector. **C** CCK-8 assay was used to detect the effects of miR-503-5p on the viability of NPCs. **D** Flow cytometry was used to detect the effects of miR-503-5p on the apoptosis rate of NPCs. **E–F** NPCs were treated with negative control miRNA (NC), PPI, IL-1β, IL-1β + PPI, and IL-1β + PPI + miR-503-5p mimics. **E** miR-503-5p mRNA in NPCs was examined through qRT-PCR. **F** Apoptosis rate was determined via flow cytometry (n = 1–2 IVDD patients/group with 3–4 samples/patient). Data are shown as means plus or minus a standard deviation. ns: not significant, *P < 0.05, **P < 0.01, and ***P < 0.001

The relation of miR-503-5p with PPI function was further analyzed. The upregulation of miR-503-5p level caused by IL-1β was decreased after treatment of PPI. The results showed that PPI could downregulate miR-503-5p expression (Fig. 2A). In order to determine the biological role of miR-503-5p in NPCs. miR-503-5p mimic was introduced to upregulate miR-503-5p expression. qRT-PCR analysis was employed to check the overexpression efficiency (Fig. 2B). Cell viability was suppressed, while the apoptosis rate was enhanced in the miR-503-5p mimics group compared with the mimics NC group (Fig. 2C, D). As shown in Fig. 2E, the expression of miR-503-5p in the IL-1β group was significantly increased compared with the NC group, whereas PPI treatment inhibited the expression of miR-503-5p, and this inhibitory effect was reversed by overexpression of miR-503-5p. In addition, flow cytometric Annexin V/PI showed that the apoptotic rate of NPCs in the IL-1β group was significantly increased compared with the NC group, whereas PPI treatment inhibited NPCs apoptosis, and this inhibitory effect was reversed by overexpression of miR-503-5p (Fig. 2F). The outcomes implicated that PPI inhibited IL-1β-induced apoptosis via the downregulation of miR-503-5p expression.

### miR-503-5p was a negative target of Bcl-2

**Fig. 3:**
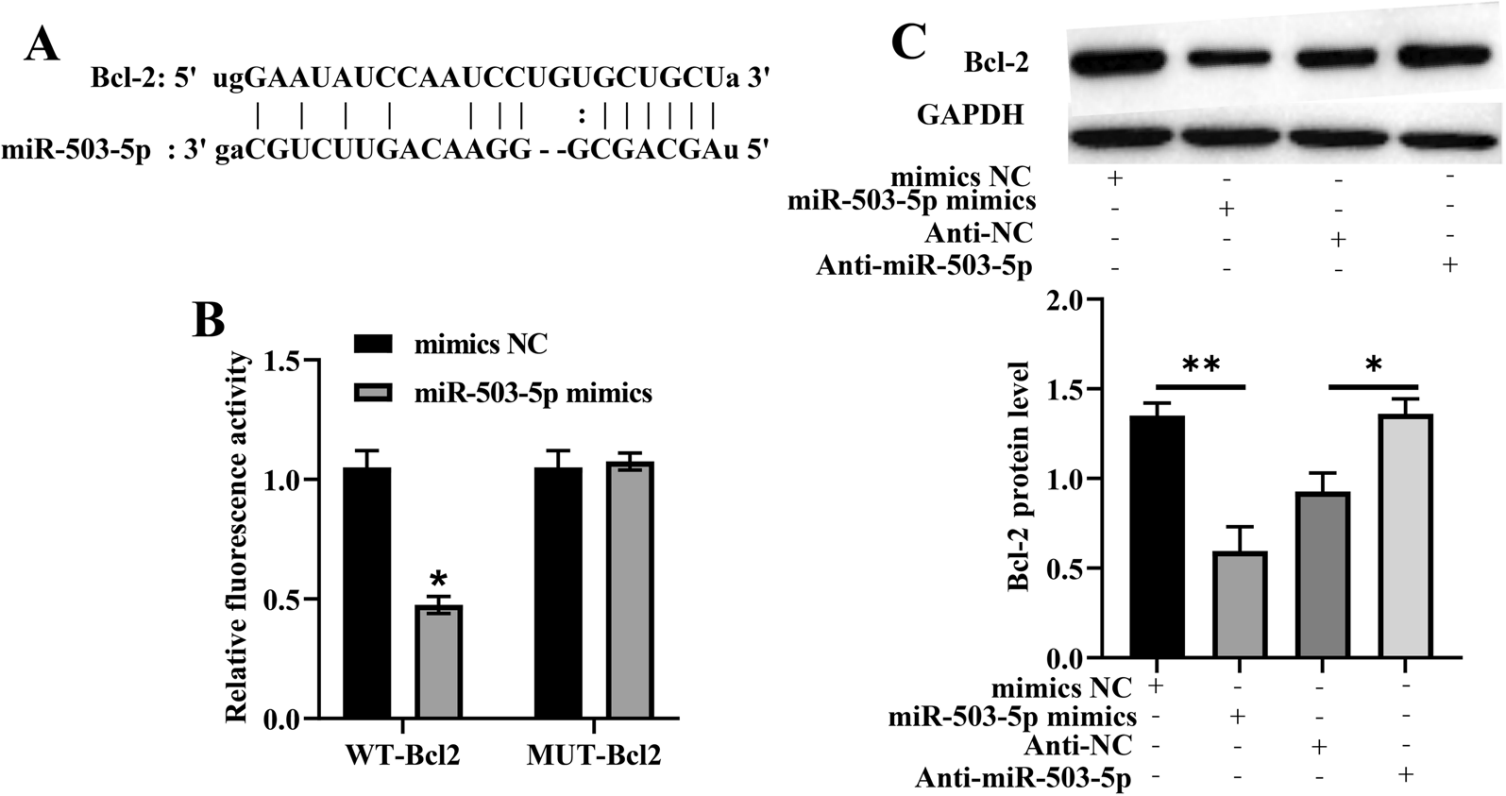
miR-503-5p was a negative target of Bcl-2. **A** ENCORI was used to predict the binding site of miR-503-5p and Bcl-2. **B** Dual-luciferase reporter gene assay was used to confirm the relationship between miR-503-5p and Bcl-2. **C** Western blot was used to detect the expression of Bcl-2 in NPCs after miR-503-5p was modulated (n = 1–2 IVDD patients/group with 3–4 samples/patient). Data are shown as means plus or minus a standard deviation (SD). ns: not significant, *P < 0.05, **P < 0.01, and ***P < 0.001

Through analyzing the ENCORI database, we found that there was a binding site in Bcl-2 3’UTR to miR-503-5p (Fig. 3A), which was consistent with the previous report [17]. Dual-luciferase reporter gene assay indicated that miR-503-5p overexpression markedly repressed the luciferase activity of Bcl-2-WT reporter but did not exert an impact on that of Bcl-2-MUT reporter (Fig. 3B). The miR-503-5p mimics significantly inhibited Bcl-2 protein expression levels, while miR-503-5p inhibitors worked oppositely. Meanwhile, there was a negative correlation between the expression levels of miR-503-5p and Bcl-2 in NPCs (Fig. 3C).

### PPI dramatically inhibited the apoptosis of NPCs induced by IL-1β via modulating miR-503-5p/Bcl-2 molecular axis

**Fig. 4:**
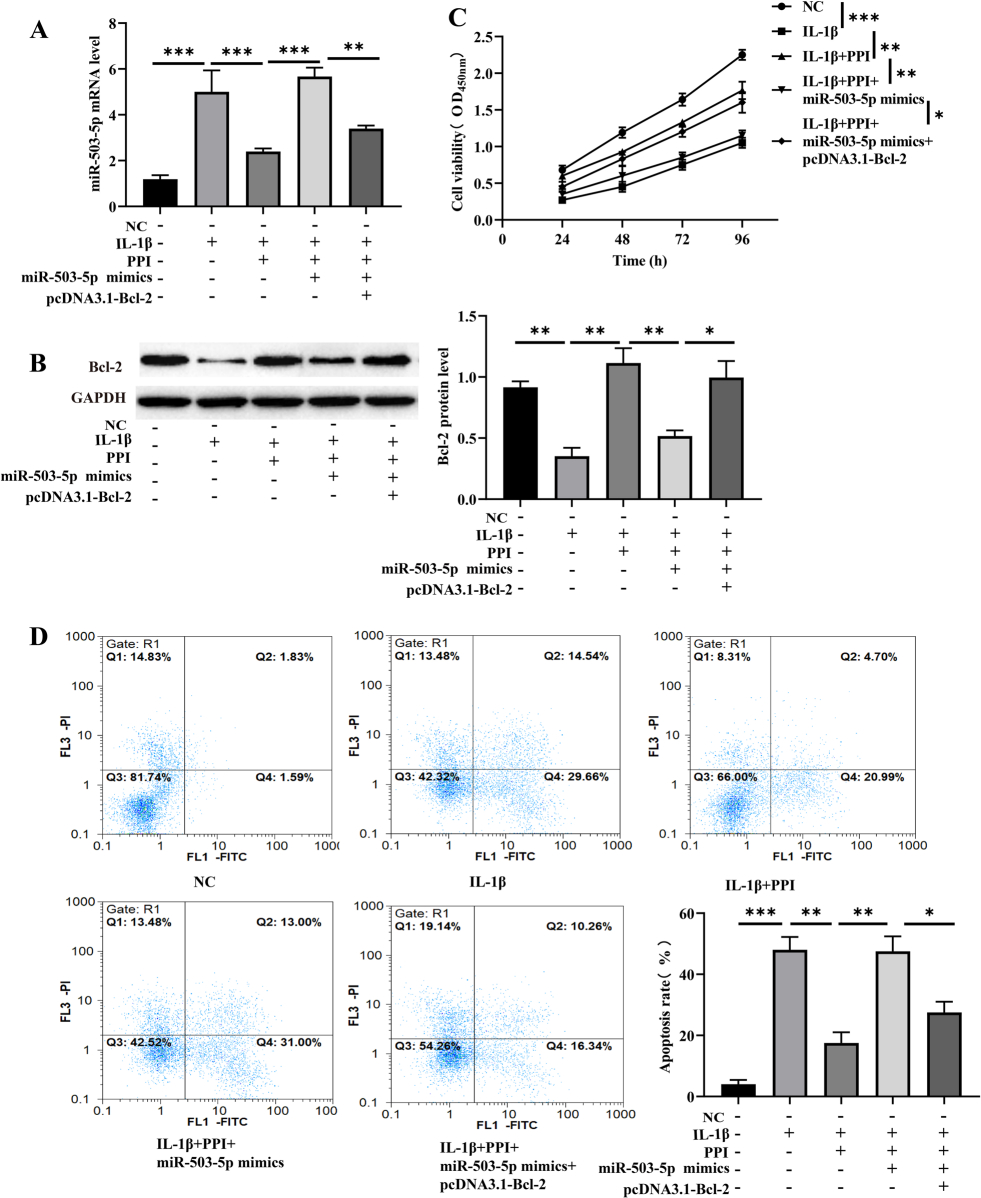
PPI inhibited the apoptosis of NPCs induced by IL-1β via modulating miR-503-5p/Bcl-2 molecular axis. **A-B** NPCs were divided into five groups: NC group, IL-1β group, IL-1β + PPI group, IL-1β + PPI + miR-503-5p overexpression group, and IL-1β + PPI + miR-503-5p overexpression + Bcl-2 overexpression group. qRT-PCR and Western blot assay were used to detect miR-503-5p mRNA and the expression of Bcl-2 in NPCs, respectively. **C** CCK-8 assay was used to detect the viability of NPCs. **D** Flow cytometry was used to detect the apoptosis rate of NPCs (n = 1–2 IVDD patients/group with 3–4 samples/patient). Data are shown as means plus or minus a standard deviation. ns: not significant, *P < 0.05, **P < 0.01, and ***P < 0.001

The rescue assays were performed to pinpoint whether PPI exerted its functions via modulating the miR-503-5p/Bcl-2 pathway. The NPCs were divided into five groups: NC group, IL-1β group, IL-1β + PPI group, IL-1β + PPI + miR-503-5p overexpression group, and IL-1β + PPI + miR-503-5p overexpression + Bcl-2 overexpression group. qRT-PCR and Western blot assay showed that PPI significantly inhibited the expression of miR-503-5p while promoting the expression of Bcl-2 in NPCs, the co-transfection of miR-503-5p reversed these effects, and the transfection of Bcl-2 overexpression plasmids counteracted the effects of miR-503-5p (Fig. 4A, B). Furthermore, functional assays showed that PPI promoted the viability and inhibited the apoptosis of NPCs; however, miR-503-5p mimics totally reversed these effects. Additionally, Bcl-2 overexpression reversed the effects induced by miR-503-5p overexpression (Fig. 4C, D). These findings demonstrated that PPI suppressed IVDD via modulating the miR-503-5p/Bcl-2 pathway.

## Discussion

In this study, we first confirmed that IL-1β induces injury in NPCs by decreasing cell viability and increasing apoptosis. We also found that IL-1β upregulated miR-503-5p in NPCs. Moreover, PPI promoted the viability and inhibited the apoptosis of NPCs. Next, dual-luciferase reporter gene assay indicated that miR-503-5p was a negative target of Bcl-2. Finally, the rescue assays were performed to pinpoint that PPI dramatically inhibited the apoptosis of NPCs induced by IL-1β via modulating miR-503-5p/Bcl-2 molecular axis.

Numerous patients are affected by IVDD, the condition that leads to low back pain and related complications, seriously damaging the labor force and bringing a heavy economic burden to society [18, 19]. Current treatments for IVDD only temporarily relieve pain, failing to eradicate the causes, and resulting in a series of sequelae [20]. The main reason for this lack of treatment is the fact that the pathogenesis of IVDD is not completely clear. Even though an increasing number of scientists are working in this field, the treatment for IVDD has not been greatly improved. To identify safer and more effective treatments for IVDD, the latest studies focused on the cellular and molecular (pathological) mechanisms related to IVDD [21, 22]. It is widely accepted that apoptosis plays an important role in the occurrence and aggravation of IVDD [23, 24]. Furthermore, the aberrant expression of IL-1β in degenerated IVDD, as well as its strong pro-apoptosis properties, has been confirmed by many studies [25, 26]. Here, to induce IVDD in vitro, we treated cells with IL-1β, similar to previous studies [27–29]. Since IL-1β induces NPCs apoptosis, the inhibition of IL-1β-induced NPCs apoptosis would be an effective treatment of IVDD [30].

PPI, one of the most important components of Rhizoma Paridis, has been proven to be safe and well-tolerated and has been widely used by clinicians in China since ancient times [31, 32]. PPI has a good anti-apoptosis effect and anti-tumor activity in previous studies [14, 33]. In this study, to mimic IVDD in vitro, we stimulated NPCs with IL-1β and then treated these cells with PPI. CCK-8 results demonstrated that PPI markedly promoted the survival rate of NPCs, suggesting that PPI effectively protect NPCs against IL-1β in vitro. We also evaluated the effect of PPI on NPCs apoptosis. Compared with the IL-1β group, the expression of pro-apoptotic genes (cleaved caspase-3 and Bax) was downregulated in the PPI treatment group. The results of flow cytometry further confirmed that PPI could alleviate NPCs apoptosis induced by IL-1β.

MicroRNAs (miRNA) are a class of small single-strand non-coding RNA molecules about 18–25 nt and play critical regulatory roles in cell differentiation, proliferation and survival [34]. In recent years, miRNA is shown to involve in the development and progression of IVDD, primarily via affecting cell apoptosis, inflammatory signal response, and ECM components. For example, miRNA-199a-5p accelerates NPCs apoptosis and IVDD by inhibiting SIRT1-mediated deacetylation of p21 [35]. miR-155 is one of the well-documented miRNAs involved in regulation of apoptotic pathways and immunological responses [36]. Liu et al*.* discovered that expression of miR-27a is high in degenerative NPCs [37]. These studies have confirmed that miRNAs promote IVDD progression by inducing apoptosis of NPCs especially miR-503-5p. It has been reported that there are 17 miRNAs abnormally expressed in the anterior cruciate ligament of degenerative disease [38], especially miR-503-5p compared with that of normal people [39]. Therefore, we attempted to investigate whether PPI protects NPCs by inhibiting the expression of miR-503-5p. Our results showed that compared with the IL-1β group, PPI treatment significantly reduced the expression of miR-503-5p in response to IL-1β stimulation, which however could be reversed by the overexpression of miR-503-5p. The results of flow cytometry further confirmed that PPI protected NPCs by inhibiting the expression of miR-503-5p. Collectively, these results indicated that PPI dramatically inhibited IL-1β-induced NPCs apoptosis via downregulating the expression of miR-503-5p.

Using the target gene prediction website, Bcl-2 was turned out to be a target gene of miR-503-5p. miR-503-5p expression was found negatively correlated with the Bcl-2 expression in our research, and the dual-luciferase reporter gene assay confirmed Bcl-2 to be a target gene of miR-503-5p. At the same time, downregulation of miR-503-5p can inhibit hepatocellular apoptosis by targeting Bcl-2 [40]. All these findings suggested that miR-503-5p can regulate the expression of its target gene Bcl-2 to promote the progression of IVDD. In addition, we also found PPI can elevate Bcl-2 protein, which however could be reversed by the overexpression of miR-503-5p, and then could be reversed by the overexpression of Bcl-2. The results of CCK-8 and flow cytometry further confirmed that PPI dramatically inhibited the apoptosis of NPCs induced by IL-1β via miR-503-5p/Bcl-2 axis. These data may provide reliable experimental basis and theoretical support for the treatment of IVDD.

## Conclusions

To sum up, we demonstrated that PPI improved cell viability and alleviated IL-1β-induced apoptosis in NPCs via modulating miR-503-5p/Bcl-2 molecular axis, providing molecular evidence that PPI can be effective on the treatment of IVDD. However, other underlying mechanisms by PPI inhibits the apoptosis of NPCs need to be further explored.

## Data Availability

The authors confirm that the data supporting the findings of this study are available within the article.

## Abbreviations

PPI: Polyphyllin I
NP: Nucleus pulposus
NPCs: Nucleus pulposus cells
IVDD: Intervertebral disk degeneration
ECM: Extracellular matrix
FBS: Fetal bovine serum
PBS: Phosphate-buffered saline
CCK-8: Cell Counting Kit-8
ND: Normal disk
SD: Scoliotic disk
